# Real-Time Lung Tumor Tracking Using a CUDA Enabled Nonrigid Registration Algorithm for MRI

**DOI:** 10.1109/JTEHM.2020.2989124

**Published:** 2020-04-24

**Authors:** Nazanin Tahmasebi, Pierre Boulanger, Jihyun Yun, Gino Fallone, Michelle Noga, Kumaradevan Punithakumar

**Affiliations:** 1 Department of Radiology and Diagnostic ImagingUniversity of Alberta 3158 EdmontonABT6G 2R3Canada; 2 Servier Virtual Cardiac CentreMazankowski Alberta Heart Institute 103114 EdmontonABT6G 2B7Canada; 3 Department of Computing ScienceUniversity of Alberta 3158 EdmontonABT6G 2R3Canada; 4 Medical Physics DivisionDepartment of OncologyUniversity of Alberta 3158 EdmontonABT6G 2R3Canada

**Keywords:** Non-rigid image registration, image segmentation, tumor tracking, radiation therapy, lung mobile tumors, GPU computing, compute unified device architecture, parallel computing

## Abstract

Objective: This study intends to develop an accurate, real-time tumor tracking algorithm for the automated radiation therapy for cancer treatment using Graphics Processing Unit (GPU) computing. Although a previous moving mesh based tumor tracking approach has been shown to be successful in delineating the tumor regions from a sequence of magnetic resonance image, the algorithm is computationally intensive and its computation time on standard Central Processing Unit (CPU) processors is too slow to be used clinically especially for automated radiation therapy system. Method: A re-implementation of the algorithm on a low-cost parallel GPU-based computing platform is utilized to accelerate this computation at a speed that is amicable to clinical usages. Several components in the registration algorithm such as the computation of similarity metric are inherently parallel which fits well with the GPU parallel processing capabilities. Solving a partial differential equation numerically to generate the mesh deformation is one of the computationally intensive components which has been accelerated by utilizing a much faster shared memory on the GPU. Results: Implemented on an NVIDIA Tesla K40c GPU, the proposed approach yielded a computational acceleration improvement of over 5 times its implementation on a CPU. The proposed approach yielded an average Dice score of 0.87 evaluated over 600 images acquired from six patients. Conclusion: This study demonstrated that the GPU computing approach can be used to accelerate tumor tracking for automated radiation therapy for mobile lung tumors. Clinical Impact: Accurately tracking mobile tumor boundaries in real-time is important to automate radiation therapy and the proposed study offers an excellent option for fast tumor region tracking for cancer treatment.

## Graphical abstract

We present the estimation of 3D left ventricular (LV) motion using the fusion of different 2D cine magnetic resonance (CMR) sequences acquired during routine imaging sessions. We show that fusing the information from short and long-axis views of CMR improves the accuracy of cardiac tissue motion estimation.

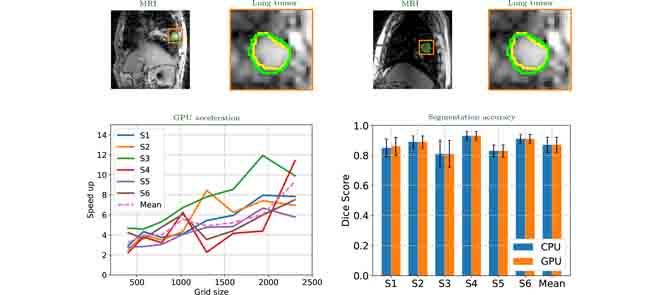

## I. Introduction

Tracking mobile tumors is crucial in the treatment of cancer patients using radiation therapy. Recently a hybrid radiotherapy MR-system, called Linac-MR, that allows for real-time MRI-guided radiation therapy with excellent soft tissue contrast for imaging tumors has been proposed [1]. The Linac-MR system also allows for real-time adjustment of the radiation beam and can be used for the therapy, given the location of the tumor is tracked over time. One approach to track mobile tumors is to find the point correspondence over a sequence of MR images acquired over a period of time. Due to the nature of non-rigid deformation of lung tissues over breathing, a diffeomorphic based non-rigid registration algorithm has been shown to be effective in accurately tracking the tumor boundaries. The non-rigid registration algorithm consists of several computationally intensive components which include geometric transformations, similarity metric, and optimization. Additionally, the registration algorithm takes many iterations to reach the final solution. Therefore, the standard Central Processing Unit (CPU) based implementation of the algorithm is time-consuming and limits its clinical application.

A few approaches have been proposed recently in the literature to track tumors from dynamic images. For instance, Zachiu *et al.* proposed an approach to track tumors from livers and kidneys for MR-guided radiation therapy using an optical tracking approach [2]. Additionally, Wilms *et al.* proposed a deformable registration approach to tackle the problem of tracking tumors from thoracic/abdominal MR and liver ultrasound data sets [3].

With the advent of NVIDIA Corporation’s Compute Unified Device Architecture (CUDA) platform, the parallel computational capabilities of Graphics Processing Units (GPUs) have become available for low-cost general purpose scientific computing. Designed originally for graphics processing, the GPUs are equipped with thousands of processing units which allows for massively parallel processing. CUDA programming model allows for thousands of concurrent threads to be executed on numerous arithmetic logical units (ALU) which leads to massive parallel execution. The reader is referred to the CUDA programming model in [4] for more detailed information about GPU programming. Recently, GPUs have been proposed to accelerate the performance of image registration algorithms [5], [6]. In addition, parallel algorithms have been proposed to process large number of data created in medical and hospital environments [7]–[9]. An optimal GPU implementation of a serial algorithm requires careful utilization of shared memory and threads management of the bottlenecks caused by memory latency and kernel execution.

This study proposes a parallel version of the moving mesh algorithm for lung tumor boundary tracking presented in [10]. Utilizing shared memory and other GPU computational resource, the proposed parallel implementation offers a speedup of more than 5 times than the CPU version allowing for the real-time application necessary in adaptive radiotherapy treatments.

## II. Registration Method

The objective of the registration approach is to find the point correspondence between two arbitrary images $T_{k_{1}}$ and $T_{k_{2}}$ defined over $\Omega \subset \mathbb {R}^{2}$. The problem can be formulated as the minimization of the $L_{2}$-norm based dissimilarity measure [11]:

\begin{equation*} \hat {\phi }_{k_{1},k_{2}} = \mathrm {arg} \underset {\phi }{\mathrm {min}}\; E_{L_{2}}(T_{k_{1}},T_{k_{2}},\phi (\xi)) \tag{1}\end{equation*}

where $\xi \in \Omega $ denotes the pixel location in the image domain $\Omega $, and the transformation function is denoted by $\phi: \Omega \rightarrow \Omega $. The dissimilarity metric based on $L_{2}$-norm is denoted by $E_{L_{2}}(\cdot)$. This formulation leads to an ill-defined problem without additional constraints and may not have a unique solution. In order to obtain a unique solution, we introduce additional parameters, namely, a Jacobian transformation $\mu:\Omega \rightarrow {\mathbb R}$ and curl of end velocity field $\gamma:\Omega \rightarrow {\mathbb R}$ to define a deformation field,

### A. Moving Mesh Generation

Let us define a continuous monitor function $\mu (\xi)$ that is constrained by:

\begin{equation*} \int _\Omega {\mu } = |\Omega |.\tag{2}\end{equation*}

The objective is to find a transformation $\phi:\Omega \rightarrow \Omega, \partial \Omega \rightarrow \partial \Omega $, so that:

\begin{equation*} J_\phi (\xi) = \mu (\xi), \tag{3}\end{equation*}

where $J_\phi $ is the Jacobian transformation.

By solving ordinary differential equation (4) and setting $\phi (\xi)=\psi (\xi,t=1)$, we could obtain a transformation function $\phi $ that satisfies (3):

\begin{equation*} \frac {d \psi (\xi,t)}{dt} = \nu _{t}(\psi (\xi,t)), \quad t \in [{0,1}], ~\psi (\xi,t=0)=\xi \quad \tag{4}\end{equation*}

where $\nu _{t}(\xi)$ is given by

\begin{equation*} \nu _{t}(\xi) = \frac {\rho (\xi)}{t+(1-t)\mu (\xi)}, \qquad t \in [{0,1}], \tag{5}\end{equation*}

for an artificially introduced algorithmic time $t$, and $\mathrm {div}\: \rho (\xi)$

\begin{equation*} \mathrm {div}\: \rho (\xi) = \mu (\xi) - 1. \tag{6}\end{equation*}

The above optimization problem may not lead to a unique solution. Therefore, we add additional constraints as below.

\begin{align*}& \mathrm {div}\; \rho (\xi) = \mu (\xi) - 1\tag{7a}\\[-0.5em]\smash {\left \{{\vphantom {\begin{matrix}.\\.\\.\\.\\ \end{matrix}}}\right.}& \\[-0.5em]& \mathrm {curl}\; \rho (\xi) = \gamma (\xi)\tag{7b}\end{align*}

with null boundary condition $\rho (\xi) = 0\,\forall \,\xi \in \partial \Omega $, where $\gamma (\xi)$ is a continuous function over $\Omega $. We then solve the resulting div-curl system under Dirichlet boundary conditions to obtain a unique solution [12]. The derivation and CPU implementation details of the algorithm could be found in [11].

This study proposes to use a GPU with shared memory to accelerate the registration algorithm through a parallel implementation of the transformation, optimization, and similarity measure components of the method. The Numbapro Python module (Continuum Analytics Inc., Austin, TX, USA) was used for the implementation of the parallel algorithm.

#### 1) Selection of Best Frame From Pretreatment Images

In Linac-MR system, the images are acquired during pretreatment for planning the radiation therapy. We utilize the pretreatment images to select the best image for every frame that we acquire during the treatment stage based on $L_{2}$-norm between the images. Let $N_{q}$, typically equal to 30, be the number of frames in the pretreatment images. The best frame $T_{q}$ (among $T_{j}$ for $j \in (1, \ldots, N_{q})$) for any image in the treatment frame $T_{k}$ can be found by:

\begin{equation*} T_{q} = \arg \min _{T_{j}} E_{L_{2}}(T_{j}, T_{k}).\tag{8}\end{equation*}

We rely on the point correspondence between $T_{j}$ and $T_{k}$ to track the tumor boundary on $T_{k}$.

Our previous study shows that using the best image based approach outperforms the approach using only the first frame in terms of accuracy and convergence [10].

## III. GPU Architecture and CUDA Implementation

The GPUs are capable of executing several threads in parallel. These threads are grouped into blocks which are executed simultaneously on streaming multiprocessors (SM). A GPU parallelization is achieved by executing a kernel function composed of multiple blocks.

The device memory, the memory space corresponds to the GPU, is physically separated from the host memory, the memory space corresponds to the CPU. The GPU arithmetic unit does not have direct access to the host memory, and therefore, the data need to be transferred to the device memory for GPU computing. At the end of the processing, the contents of the device memory will be transferred back to the host memory so that the CPU can access the final results.

GPU memory space is further divided into global memory, shared memory, and registers. These memory units differ in terms of access speed, memory size, and data accessibility.

1) Global memory can be accessed by any thread running on the GPU. It is the memory used for transferring data from the host computer. GPU global memory has slower access speed in comparison to shared memory and registers. The typical access latency for global memory is in the order of 400–600 GPU clock cycles [13].
2) The GPU shared memory unit has much faster access speed than the global memory (almost 100 times). However, the shared memory is only accessible with the threads of a block, and the threads from a different block cannot access the data stored in the memory. Additionally, the data to the shared memory cannot be transferred directly from the host memory and need to be transferred first to the GPU global memory.

Further details about the GPU architecture could be found in the CUDA Programming Guide [14]. In this study, we utilize the improved access speed of the shared memory of the GPU to accelerate the numerical computations of the partial differential equation, (4), where the data is accessed multiple times by the GPU ALUs.

## IV. Parallel Implementation of the Mesh Algorithm

**FIGURE 1.**
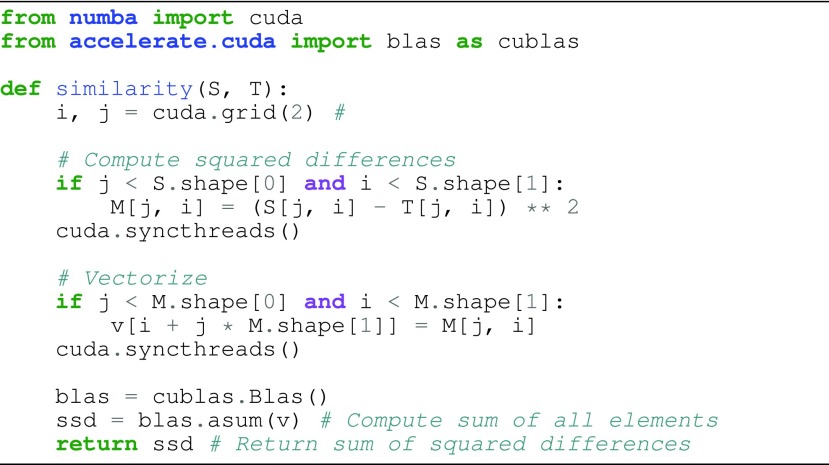
The code snippet showing the parallel implementation of the similarity metric computation using numba and accelerate Python modules.

The proposed nonrigid registration approach consists of moving mesh generation, $L_{2}$-norm based dissimilarity metric computation, optimization and transformation. All of the individual components of the registration approach are parallelized and performed on the GPU [15]. An example code snippet to compute the dissimilarity metric is given in Fig. 1. A similar parallelization approach is pursued for other computational components of the registration algorithm. The GPU hardware allows for executing a large number of parallel threads simultaneously. In GPU computing, a group of threads that are executed together are called blocks and a group of blocks are called grid. The optimum number of blocks per thread was computed based on the size of the moving mesh. The transformation computation for each point on the moving mesh is computed in parallel. The data transfer from host to device memories and device to host memories were performed only once to reduce data transfer overhead. A single-precision floating point data type was used for the GPU implementation.

An NVIDIA Tesla K40c based on the Kepler™ Architecture was used to test our implementation [16]. The Tesla K40c is a professional grade graphics card which supports double precision computing. It has been built on the 28 nm process and based on the GK110B graphics processor architecture which has a large chip with a die area of $561~mm^{2}$ and 7,080 million transistors. The Tesla K40c consist of 15 SMs each featuring 192 single-precision CUDA cores, totaling 2880 CUDA cores. Each SM uses four warp schedulers and eight instruction dispatch units and equipped with 1.5 MB of L2 cache, 64 KB of constant memory and 48 KB of shared memory. The graphics card has 12,288 MB GDDR5 memory which is connected using a 384-bit memory interface with a maximum clock speed of 3.00 GHz and 288 GB/sec of memory bandwidth. The transfer of data between host (CPU) and device (GPU) is achieved through a PCIe-3 Bus.

## V. Experimental Protocol

The experimental protocol was approved by the University of Alberta Health Research Ethics Board. The data was acquired from six lung cancer patients with free breathing using a 3T MRI scanner. In order to simulate the actual scanning quality reduction due to the 0.5T MRI used in adaptive radiation therapy, additional noise is added to the MR image data. Each patient data consists of 30 pretreatment images and 100 images correspond to the treatment stage. The pretreatment and treatment stages will be performed using the same MR protocol using the linac-MR [17] in real-time scanning and radiation treatment. The selection of the MR imaging plane is based on the view of the maximum tumor dimension for the beam’s eye view. The acquisition rate of the MRI is 4 frames per second. Table 1 reports the details of the data sets used in this study. The ground truth manual segmentation of the tumor region is performed by an expert radiation oncologist. The CPU version of the algorithm uses a single-thread implementation which was evaluated on a 2.6 GHz Intel Core i7 processor. The CPU implementation primarily relied on the Numpy Python module for the computation of similarity metric, transformation, optimization and other operations. One of the computationally intensive task related to the tri-linear interpolation was implemented using Cython to accelerate the computational performance.

**TABLE 1:** Details of the Datasets Used in Evaluation of the Proposed Method

Description	Value
Number of subjects	6
Breathing pattern	free breathing
Number of pretreatment frames	30 per subject
Number of treatment frames	100 per subject
Original image matrix	$128 \times 128$ pixels
Reconstructed image matrix	$256 \times 256$ pixels
Field of view	$400\times 400\,\,mm$
Pixel spacing	$1.56 \times 1.56\,\,mm$
Imaging speed	4 fps
TE	1.1 ms
TR	2.2 ms
Slice orientation	sagittal
Slice thickness	20 mm

### A. Evaluation Criteria

Quantitative evaluations to measure the similarities between manual delineation and automated segmentation by GPU and CPU approaches were performed using Dice Metric (DM) [10], Hausdorff Distance (HD) [10] and Root Mean Square Error (RMSE) [10].

## VI. Results

### A. Quantitative Evaluation

The performance of the best frame approach in terms of DM, HD and RMSE for the CPU and GPU implementation is reported Table 2. The corresponding results are also displayed in Fig. 3. Both GPU and CPU based methods yielded an average DM of 0.87 and average RMSE of 1.48 mm. Table 3 shows mean and standard deviation of centroid difference in superior–inferior (S–I) and anterior–posterior (A–P) directions as well as 2-dimensional centroid difference for each subject in the GPU and CPU implementation. The corresponding results are also displayed in Fig. 4.

**TABLE 2:** Mean and Standard Deviation of Dice Metric, Hausdorff Distance and RMSE for Each Patient for CPU and GPU Implementations in Comparison to Expert Manual Contours. Both Methods Yielded the Similar Level of Segmentation Accuracy

	CPU			GPU		
Subject	DM (SD)	HD (SD) mm	RMSE (SD) mm	DM (SD)	HD (SD) mm	RMSE (SD) mm
Subject 1	0.85(0.06)	3.98(1.38)	1.68(0.61)	0.86(0.06)	3.90(1.34)	1.65(0.59)
Subject 2	0.89(0.04)	3.67(1.12)	1.52(0.51)	0.89(0.04)	3.69(1.13)	1.54(0.52)
Subject 3	0.81(0.09)	3.44(1.35)	1.46(0.64)	0.81(0.09)	3.47(1.36)	1.46(0.64)
Subject 4	0.93(0.03)	2.86(0.74)	1.17(0.34)	0.93(0.03)	2.87(0.75)	1.16(0.34)
Subject 5	0.83(0.04)	4.23(1.75)	1.45(0.45)	0.83(0.04)	4.23(1.75)	1.46(0.45)
Subject 6	0.91(0.03)	3.89(1.11)	1.57(0.42)	0.91(0.03)	3.88(1.12)	1.58(0.42)
Mean	0.87(0.05)	3.68(1.24)	1.48(0.50)	0.87(0.05)	3.67(1.24)	1.48(0.49)

**TABLE 3:** Mean and Standard Deviation of Centroid Difference in Superior–Inferior and Anterior–Posterior Directions as Well as 2-Dimensional Centroid Difference. The GPU and CPU Implementations Yielded Similar Level of Accuracy

	Centroid difference, CPU			Centroid difference, GPU		
Subject	$L_{2}$-norm (SD) mm	S–I (SD) mm	A–P (SD) mm	$L_{2}$-norm (SD) mm	S–I (SD) mm	A–P (SD) mm
Subject 1	1.67 (0.94)	0.97 (0.64)	1.19 (0.95)	1.62 (0.93)	0.95 (0.63)	1.17 (0.93)
Subject 2	1.52 (0.89)	0.96 (0.78)	1.00 (0.77)	1.55 (0.88)	0.96 (0.79)	1.04 (0.76)
Subject 3	1.76 (1.25)	1.17 (0.84)	1.20 (1.06)	1.75 (1.27)	1.20 (0.89)	1.16 (1.06)
Subject 4	1.13 (0.74)	0.65 (0.53)	0.82 (0.67)	1.13 (0.74)	0.66 (0.53)	0.82 (0.66)
Subject 5	2.18 (1.41)	1.45 (1.05)	1.49 (1.13)	2.14 (1.39)	1.43 (1.02)	1.46 (1.14)
Subject 6	1.46 (0.80)	1.11 (0.80)	0.72 (0.62)	1.46 (0.78)	1.10 (0.78)	0.72 (0.63)
Mean	1.62 (1.01)	1.05 (0.77)	1.07 (0.87)	1.61 (1.00)	1.05 (0.77)	1.06 (0.86)

**FIGURE 2.**
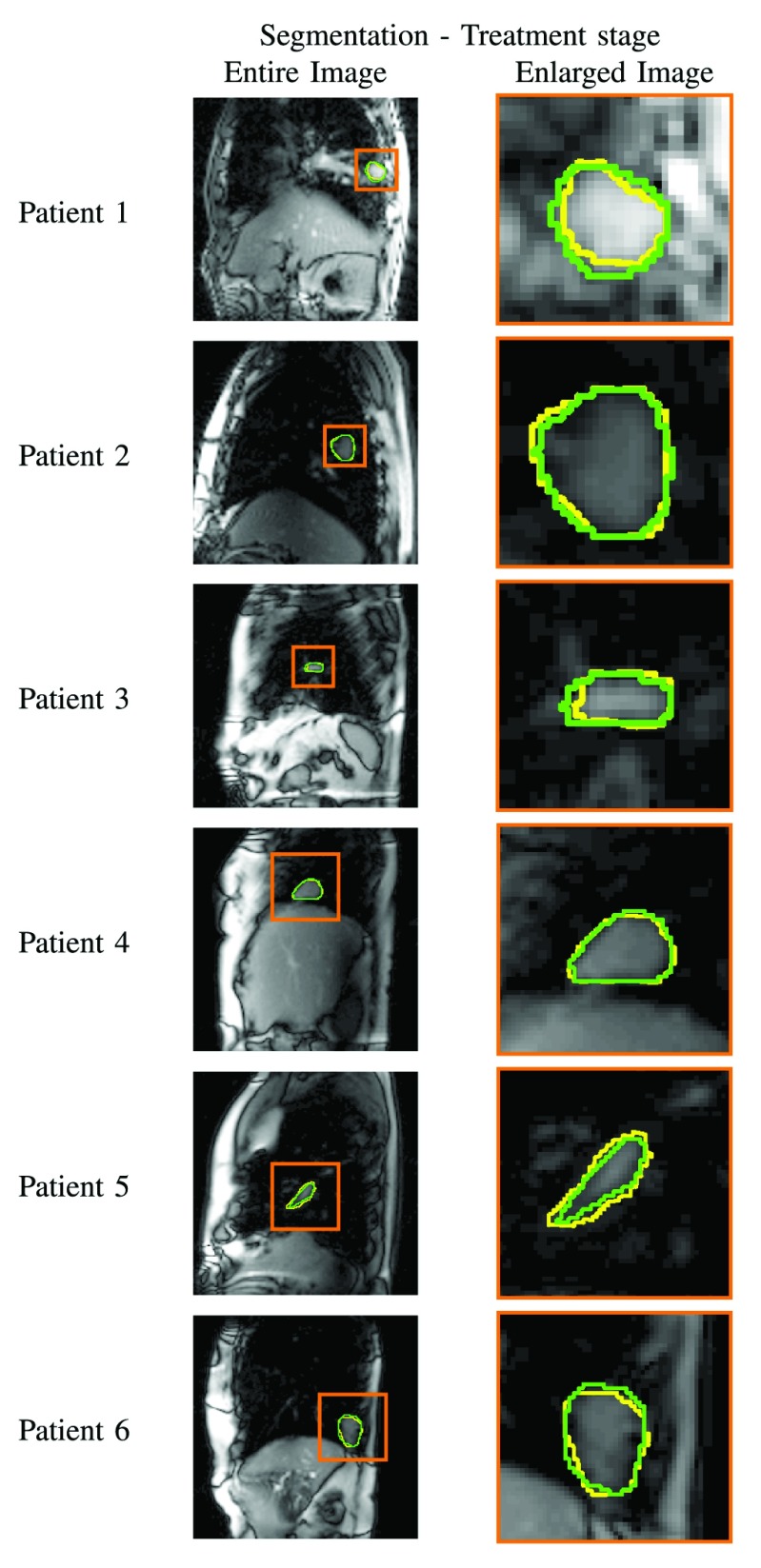
Representative examples showing the automated (yellow) and manual (green) boundaries of the tumor regions for the registration techniques for all patients.

**FIGURE 3.**
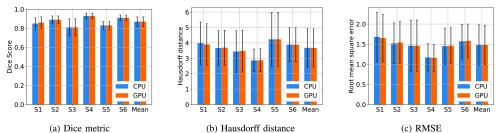
Mean of Dice metric, Hausdorff distance and RMSE for segmentation results by CPU and GPU implementation for each patient, S1 to S6, in comparison to expert manual contours. The error bar indicates the standard deviation.

**FIGURE 4.**
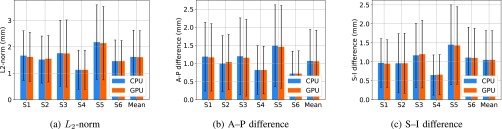
Mean centroid differences in anterior-posterior and superior-inferior directions as well as 2-dimensional centroid difference for the CPU and GPU implementation results in comparison to expert manual contours. The error bar indicates the standard deviation.

### B. Computational Efficiency

Table 4 shows the runtime of the CPU implementation of the algorithm for different grid sizes of the mesh deformation based registration algorithms for each subject. The shared memory based GPU implementation of the algorithm is evaluated over different grid sizes and the corresponding runtimes are reported in Table 5. The NVIDIA Visual Profiler was used for obtaining the precise computational times for the GPU implementation. The computational times include the data transfer between device and host memory locations. The results demonstrate that the GPU based implementation yielded an acceleration of more than 5 times in comparison to the implementation using CPU. The speedup of the GPU algorithm in comparison to the CPU version is given Table 6 and Fig. 7.

**TABLE 4:** The Runtime of the CPU Implementation of the Mesh Deformation Based Registration Algorithm for Different Grid Sizes

Grid Size pixels $\times $ pixels	Subject 1 ms	Subject 2 ms	Subject 3 ms	Subject 4 ms	Subject 5 ms	Subject 6 ms	Mean ms	SD ms
$20 \times 20$	157.00	93.00	313.00	141.00	109.00	141.00	159.00	79.02
$24 \times 24$	187.00	188.00	390.00	312.00	110.00	156.00	223.83	105.44
$28 \times 28$	188.00	188.00	483.00	281.00	125.00	188.00	242.17	128.09
$32 \times 32$	234.00	250.00	618.00	562.00	156.00	375.00	365.83	188.14
$36 \times 36$	282.00	313.00	837.00	312.00	219.00	266.00	371.50	230.67
$40 \times 40$	265.00	266.00	484.00	812.00	235.00	172.00	372.33	239.83
$44 \times 44$	360.00	283.00	438.00	1015.00	297.00	265.00	443.00	287.30
$48 \times 48$	344.00	312.00	453.00	2225.00	313.00	296.00	657.17	770.18

**TABLE 5:** The Results of GPU Implementation of the Mesh Deformation With Shared Memory on Different Grid Sizes and on a Nvidia Tesla K40c

Grid Size pixels $\times $ pixels	Subject 1 ms	Subject 2 ms	Subject 3 ms	Subject 4 ms	Subject 5 ms	Subject 6 ms	Mean ms	SD ms
$20 \times 20$	53.51	36.66	66.96	63.05	39.34	33.27	48.80	14.37
$24 \times 24$	43.16	47.01	84.98	82.02	38.77	43.23	56.53	21.07
$28 \times 28$	50.33	53.87	90.84	87.60	41.04	39.22	60.48	22.95
$32 \times 32$	56.92	57.41	91.98	89.75	38.53	61.82	66.07	20.82
$36 \times 36$	51.86	37.06	107.30	137.73	46.02	74.95	75.82	39.47
$40 \times 40$	44.46	42.52	56.63	195.38	48.60	38.95	71.09	61.19
$44 \times 44$	45.24	38.20	36.69	231.43	44.55	44.27	73.40	77.50
$48 \times 48$	43.90	44.86	45.71	195.21	54.12	39.43	70.54	61.26

**TABLE 6:** GPU Speedup Results for Each Patient for Different Grid Sizes

Grid Size pixels $\times $ pixels	Subject 1	Subject 2	Subject 3	Subject 4	Subject 5	Subject 6	Mean	SD
$20 \times 20$	2.93	2.54	4.67	2.24	2.77	4.24	3.26	5.50
$24 \times 24$	4.33	4.00	4.59	3.80	2.84	3.61	3.96	5.00
$28 \times 28$	3.74	3.49	5.32	3.21	3.05	4.79	4.00	5.58
$32 \times 32$	4.11	4.35	6.72	6.26	4.05	6.07	5.54	9.04
$36 \times 36$	5.44	8.45	7.80	2.27	4.76	3.55	4.90	5.84
$40 \times 40$	5.96	6.26	8.55	4.16	4.84	4.42	5.24	3.92
$44 \times 44$	7.96	7.41	11.94	4.39	6.67	5.99	6.04	3.71
$48 \times 48$	7.84	6.95	9.91	11.40	5.78	7.51	9.32	12.57

**FIGURE 5.**
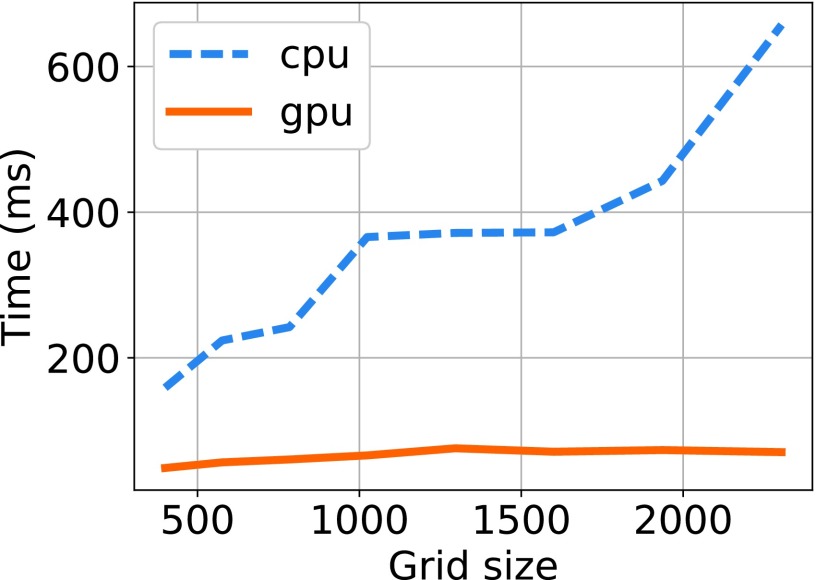
Mean computational times for GPU and CPU implementation of the algorithm over different grid sizes.

**FIGURE 6.**
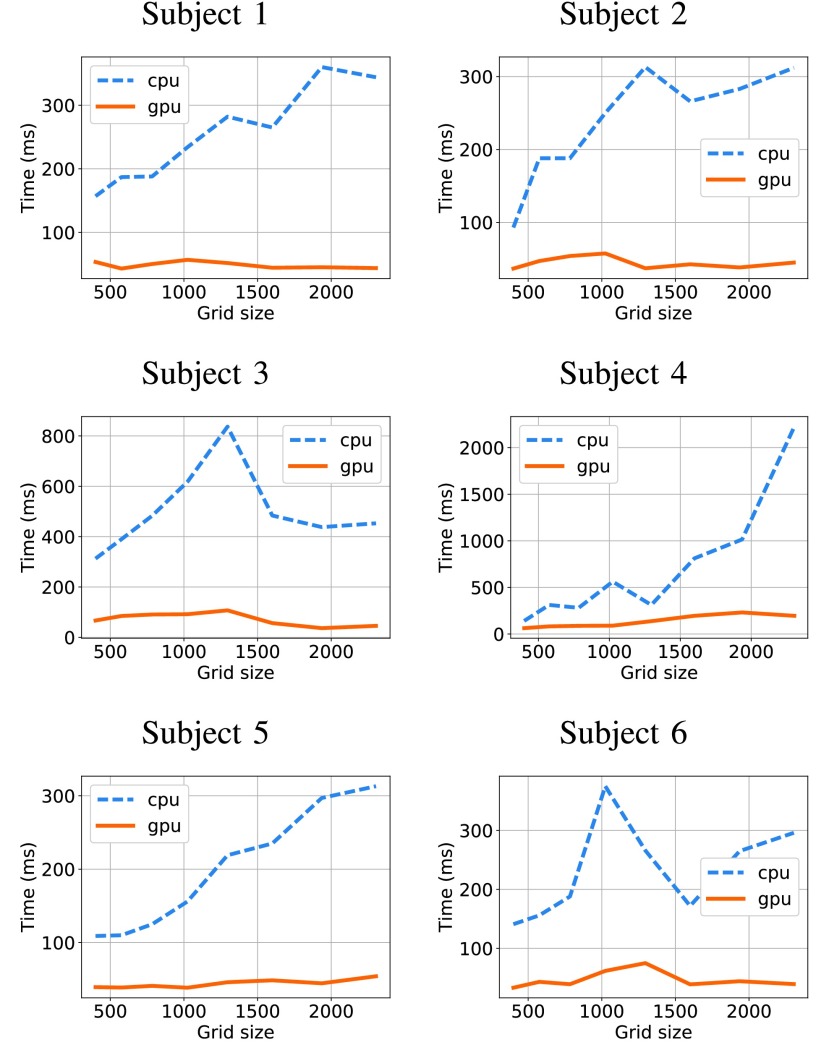
Computational times for GPU and CPU algorithms in each subject.

**FIGURE 7.**
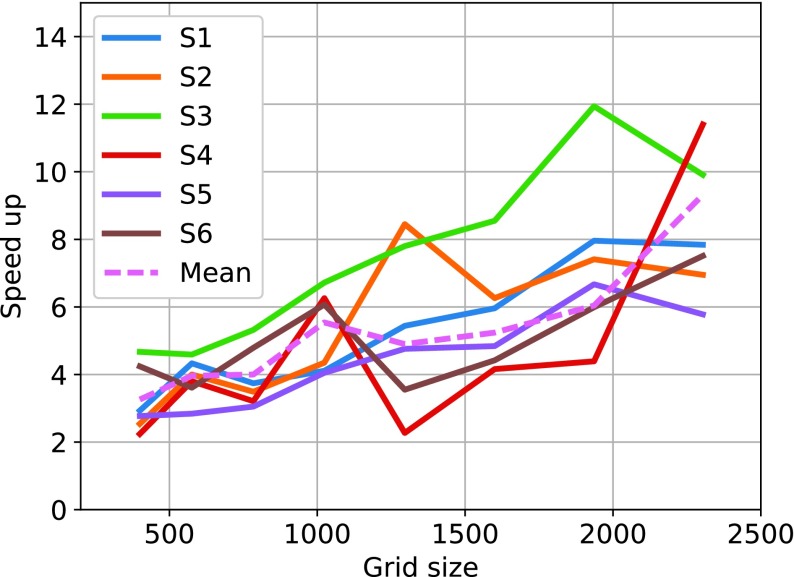
GPU speedup for different grid sizes for each patient.

Fig. 5 shows the mean computational times over the entire subject set for different grid sizes by the GPU and CPU versions of the proposed algorithm. The curves for individual subjects are give in Fig. 6.

## VII. Discussion

The usage of GPUs have been shown to be an important factor for the real-time clinical applications of the image registration algorithms [18]–[24]. Computing joint histograms on the GPU is reported in [25]–[27]. Haghighi *et al.* proposed a framework for an intensity-based symmetric registration method where the GPU implementation leads to an improved computational performance [28]. In [29], authors proposed a GPU accelerated the computation of an affine-linear transformation in a derivative-based optimization framework where the GPU approach outperformed the CPU version by more than 2 times. For more information about image registration and GPU, the reader is referred to two survey articles by Shams *et al.*, [30] and Fluck *et al.*, [5].

The parallel implementation approach proposed in this study allows for the computation of the entire registration algorithm on the GPU. In contrast, only one or more individual components were parallelized in several previous algorithms. For instance, only the dis-similarity measure was computed in the method proposed by Kubias *et al.* [18]. Ruiz *et al.* proposed computation of only the cross-correlation component using GPU computing [19]. The method by Huang *et al.* relied on CPU for the histogram and dis-similarity metric computations, and only the transformation was performed using a GPU.

The results for the CPU version of the proposed nonrigid registration algorithms on the same dataset is presented in [10]. The proposed nonrigid registration based algorithm does not utilize shape or distance priors in the segmentation process as in [31], [32].

In this study, we have shown that the same level of accuracy can be achieved for the segmentation of tumor regions using the GPU version of the algorithm. The GPU version generates nearly identical moving mesh correspondences produced by the CPU version. One of the main advantages of the GPU approach is that the computational time does not linearly increase with the size of the grid as shown in Fig. 5. Due to the large number of computing units available in GPUs such as Tesla K40c, the computational time required for the parallel execution of the data points on the grid largely depends on the number of iterations required to obtain the final solution rather than the size of the grid [33].

Beyond accelerating image processing applications, GPUs have also been applied to image reconstruction algorithms for faster processing. The accelerated computing of graphics hardware was exploited for the computed tomography (CT) imaging modality earlier than the MRI. MR images can often be reconstructed with fast Fourier transform algorithm whereas CT requires a more computationally demanding reconstruction approach [5]. One application of the GPU acceleration of MRI reconstruction is to increase the spatial and temporal resolution with compressed sensing [34].

Although the underlying anatomical motion is in three-dimensional space, the Linac-MR [1] radiotherapy system generates two-dimensional slices acquired at a planar location. Therefore, registration in two-dimensional space is adequate for the proposed problem.

## VIII. Conclusion

The study proposes an application of GPUs to accelerate the tracking of lung tumors for using non-rigid image registration algorithm. The study compares the parallel GPU implementation with shared memory optimization version of the algorithms with the traditional CPU implementation. Quantitative performance evaluations show that the GPU implementation of the algorithm yielded a computational acceleration of 5 times over the CPU implementation while retaining a similar level of segmentation accuracy.
